# Melbourne Notes

**Published:** 1909-08-14

**Authors:** 


### Melbourne Notes.

(From a Correspondent.)

The rebuilding of the Melbourne General Hospital, contemplated for the past twenty years, will be begun early in the new year. The present hospital has long been too small, and, as it is to be rebuilt on the same site, it must naturally ascend nearer the clouds. The enterprise has been facilitated by the gift of £120,000 from the Trustees of the Edward Wilson (founder of the *Argus* newspaper) Estate, September 1907. One of the conditions of the donation is that building shall be commenced within eighteen months and completed within five years. No part of the hospital will be closed, as the work will be done piecemeal, beginning at the north-west corner, where at present stands the Secretary's house, which will be demolished to make room for a much larger—and it is to be hoped much better—out-patients' department. A feature of the new hospital is to be the most up-to-date provision for clinical investigation, and it is expected that the State of Victoria will be put in possession of one of the finest hospitals in the world. In pursuance of this ideal it is proposed to create new departments—gynæcological, eye and ear, etc. This proposal brought forth strong protests from the committees and medical officers of the special hospitals. A deputation waited on the State Treasurer, who has the allocation of the Government charity money, and it was only on the solemn promise of the Melbourne Hospital representatives that these special departments would not be allowed to expand beyond their scope as observation wards that the scheme was passed, the conditions being that it should have a ten years' trial, and that only one specialist should be appointed to each department. As the Treasurer remarked, "The overlapping of our charities will have to be legislated for at an early date." He further said that he contemplated introducing legislation to control the charity system, to deal with the mode of electing medical staffs, and to establish an intermediate hospital for patients of small means. As a fact, the Melbourne Hospital is almost alone here in having no special departments, the Alfred and St. Vincent's and the Sydney General Hospitals all following the British model.

Meantime an unknown donor had offered £5,000 towards the building of a children's wing at the Homœopathic Hospital. The idea was submitted to the State Treasurer with a request for additional financial assistance. A reply in the negative was given promptly. There was already a well-equipped Children's Hospital. Some of the wards could be set apart for the homœopathic treatment of children, and the doctors of the two schools of medicine need not come into contact.

Simultaneously the Alfred, the other large general hospital of Melbourne, is about to spend the sum of £13,000 on enlargements and repairs, another indication that no matter how much hospital acommodation were provided it would all be taken up immediately.

---

Mr. A. White, a receiving clerk in the Melbourne Telegraph Department, has patented a very neat invention in connection with telephone mouthpieces—a tiny, hollow drum of antiseptic paper attached to the transmitter. The piece of paper used at each conversation is torn off and a fresh slip drawn over.

---

The rapid success which attended the publication of Dr. T. D. Savill's "System of Clinical Medicine" has rendered a second edition necessary, and the author has taken advantage of this to correct and revise the whole work throughout. The new edition will be in one volume, and will be issued by Mr. Edward Arnold about September 1.

